# Connecting the lines after a stroke

**DOI:** 10.7554/eLife.81306

**Published:** 2022-07-28

**Authors:** S Thomas Carmichael

**Affiliations:** 1 Department of Neurology, David Geffen School of Medicine at UCLA Los Angeles United States

**Keywords:** plasticity, optogenetics, stroke, functional connectivity, brain, Mouse

## Abstract

In mice, stimulating cortical areas in the undamaged hemisphere of a brain affected by stroke impairs recovery.

**Related research article** Bice AR, Xiao Q, Kong J, Yan P, Rosenthal ZP, Kraft AW, Smith KP, Wieloch T, Lee JM, Culver JP, Bauer AQ. 2022. Homotopic contralesional excitation suppresses spontaneous circuit repair and global network reconnections following ischemic stroke. *eLife* **11**:e68852. doi: 10.7554/eLife.68852.

The brain is divided into hemispheres which are connected through a bundle of nerve fibers known as the corpus callosum. Each hemisphere controls the opposite side of the body: for example, a precise cortical area in the right hemisphere will be responsible for sending and receiving information to and from the left hand. This cortical area is connected to neighboring brain territories as well as to corresponding regions in the opposite (or ‘contralateral’) hemisphere via callosal connections (Figure 1A).

**Figure 1.**
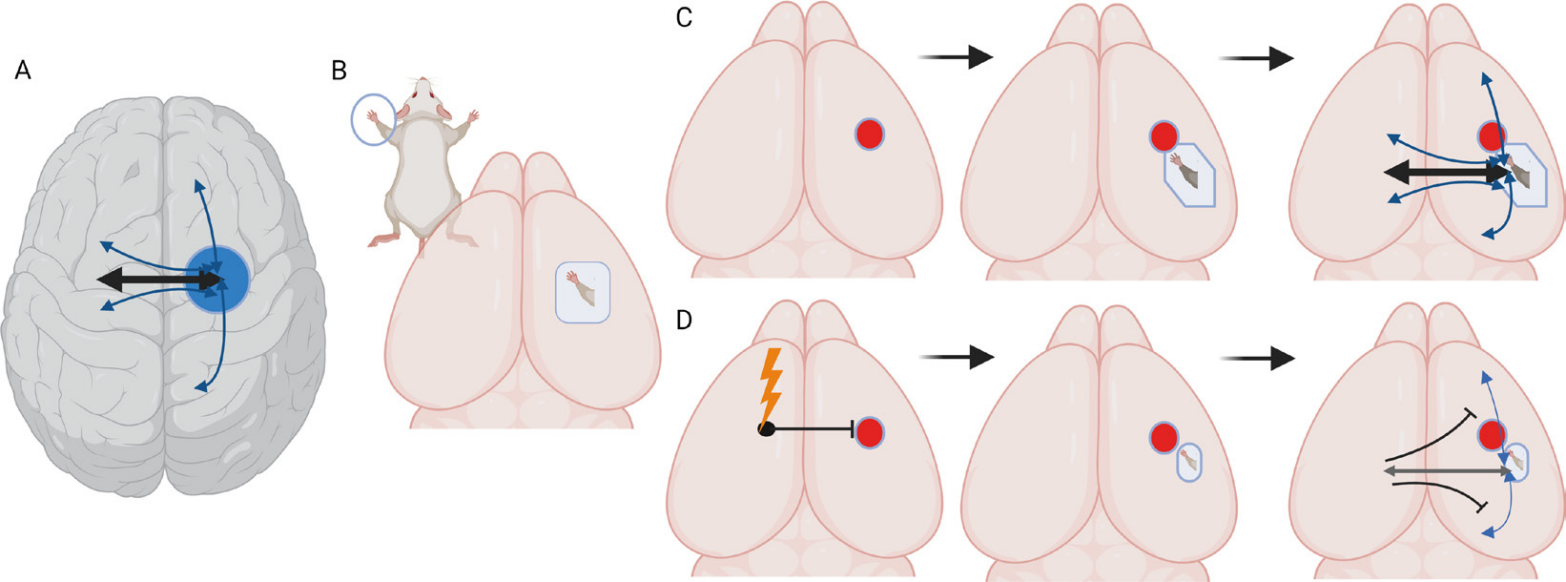
Stimulating cortex areas contralateral to the site of a stroke impairs recovery and reconnection. (**A**) Dorsal view of the human brain. Cortical areas in the human brain are functionally connected with regions in both the same (ipsilateral) and opposite (contralateral) hemisphere. These connections (represented by arrows) are present when brain areas are co-active in time and are associated with a behavioral output, such as moving or sensing the hand. (**B**) Dorsal view of the mouse brain. Stimulation of the mouse forepaw activates a specific region of the primary somatosensory cortex that the paw is mapped on to, which is the area studied by Bice et al. (**C**) Inducing a stroke in the forepaw area (red circle) stops the activation of this region and the surrounding cortex following forepaw stimulation for at least seven days. At later time points (one month), stimulation of the forepaw is once again able to activate the brain, this time in a forepaw representation that is shifted to a new site due to cortical plasticity. At later time points, the forepaw cortical area is re-integrated into a network in which it is connected to ipsilateral sensory, motor and cortical associated areas, and contralateral sensory and motor areas. (**D**) Chronic, daily stimulation of the somatosensory cortex that is contralateral to the stroke site (lightning bolt) impairs the local remapping of the forepaw area and interferes with the integration of the recovered cortex into functional brain networks. Under these conditions, callosal connections, when activated, actually inhibit large areas of the cortex in the opposite hemisphere.

Image credit: Created with biorender.com.

Electrically stimulating certain regions of the brain is relatively simple, and it has long been pursued to enhance recovery after injury or stroke (when the blood supply to a brain region stops). In healthy individuals, for example, directly stimulating motor areas improves motor skill performance (Lefaucheur, 2019). At present, 301 clinical trials are registered to explore the impact of brain stimulation on stroke patients, examining outcomes ranging from upper or lower limb function to the ability to reflect on one’s thinking. In particular, inhibiting a motor region in one hemisphere may enhance the activity and motor performance of the corresponding area in the contralateral hemisphere, potentially leading to improved motor function after a stroke. However, this contralateral inhibition approach has not been consistently effective at improving brain recovery (Henrich-Noack et al., 2017).

To develop enhanced approaches, scientists need to first have a better understanding of how stimulation impacts the circuitry which connects the brain hemispheres. Now, in eLife, Adam Bauer and colleagues at Washington University in St. Louis and Lund University – including Annie Bice as first author – report new findings that suggest stimulating cortical areas contralateral to the regions affected by a stroke inhibits recovery (Bice et al., 2022).

The team induced small strokes in an area in the cortex of mice which had been genetically engineered so that some of their excitatory neurons could be activated by light; the cortical area targeted is activated by sensory and motor information from one of the animal’s forepaws (Figure 1B). Next, specific excitatory neurons in the contralateral (non-damaged) cortex were optically stimulated for five consecutive days for four weeks. This procedure has been shown to enhance neuronal activity in connected brain areas (Cheng et al., 2014).

Following a stroke, the brain being able to exhibit plasticity – and therefore nearby cortical areas taking on the role of the damaged regions – is associated with recovery. Examining this re-mapping of cortical connections can give an independent indicator of how damaged or plastic the circuitry may be after a stroke. To explore how contralateral stimulation affected this process, Bice et al. recorded behavioral recovery and assessed the functional connectivity of cortical areas. In addition, they mapped how the forepaw was represented in the mice’s cortex using optical intrinsic signal imaging, a method based on blood flow which helps to capture the functional architecture of the cortex. This showed that one week after the stroke, the damaged brain area or its immediate surroundings exhibited no or blunted responses when the corresponding paw was stimulated. Yet, as noted by other reports, areas near the wounded region started to respond to stimulation over time, highlighting that cortical activation had spontaneously recovered (Figure 1C; Brown et al., 2009). However, Bice et al. found that chronic, daily stimulation of the contralateral cortex impaired this plasticity and the natural recovery of the cortical map after a stroke.

A stroke disconnects brain regions that normally have concerted or joint activity in mediating a particular task. These networks of co-active brain areas have already been identified in humans (Bowren et al., 2022). Here, Bice et al. harnessed optical intrinsic signal imaging to examine the state of these networks in mice. They found that following spontaneous recovery, regions in or near the stroke part of the cortex had regained highly correlated patterns of activity with their healthy counterparts on the other hemisphere. However, chronic stimulation of the cortex contralateral to the site of the stroke inhibited this re-establishment of functional connections across the hemispheres.

Finally, Brice et al. explored callosal function in healthy and stroke brains. Optically stimulating cortical regions in the healthy hemisphere and tracking their connections in awake mice showed that, as expected, callosal connections activate the regions they are tied to in the opposite hemisphere. However, they also slightly inhibit the functional connections between these regions and the areas around them. Chronically stimulating the contralateral cortex after a stroke significantly increases and extends this callosal inhibition, therefore impairing the remapping process that takes place naturally after a stroke (Figure 1D).

Certain limitations prevent the conclusions of this study from being applied more widely. For example, all excitatory neurons were stimulated and not just callosal neurons; this means that the effects observed in the damaged hemisphere may not be due to a direct relay across callosal connections, but instead because of circuit processing in the stimulated cortex or in other brain areas. In addition, it is unclear how the results could apply to human brains, for which stroke affects larger areas and involves cortical and subcortical damage (Otsuka and Kawaguchi, 2011; Petrus et al., 2020). Finally, the work establishes important data for only one type of cortical circuit, and other kinds (such as the motor cortex) may vary in their response to injury or stimulation. Nevertheless, the experiments conducted by Bice et al. pave the way for a more nuanced approach to brain stimulation for stroke recovery.
